# Validating the antiseizure effects of vitexin and related flavone glycosides in zebrafish

**DOI:** 10.3389/fphar.2025.1628324

**Published:** 2025-06-26

**Authors:** Audrey Breckenridge, Sanskriti Basnyat, Eva Fitch, Colleen Carpenter-Swanson

**Affiliations:** Department of Biology, University of Richmond, Richmond, VA, United States

**Keywords:** vitexin, flavonoids, epilepsy, zebrafish, behavior, multielectrode array

## Abstract

Current epilepsy treatments often fail to provide sufficient control over seizures, highlighting the need for new therapeutic agents. Vitexin, a flavone with antioxidant, anti-inflammatory, and neuroprotective properties, was previously shown to suppress seizure activity in rodent models. Utilizing zebrafish, this study further evaluates the antiseizure properties of vitexin and for the first time, examines the related flavone glycosides: isovitexin, vitexin 2-O-rhamnoside, vitexin-4-O-glucoside and saponarin. We initially tested the ability of the compounds to reduce behavioral seizures stimulated by the GABA_A_ receptor antagonists (pentylenetetrazole: PTZ and picrotoxin: PTX) and spontaneous seizures in a genetic epilepsy model (Dravet syndrome, *scn1lab* ^*−/−*^ zebrafish larvae). Seizure behavior was quantified in 5-day old larvae via automated tracking with a DanioVision monitoring chamber linked to EthoVision XT 15 software. Microelectrode array electrophysiology (MEA) was then used to examine the effects on PTZ-induced seizure-like brain activity. While having no effect on basal locomotion, vitexin and isovitexin significantly reduced seizure activity in PTZ-treated zebrafish. None of the flavones exhibited antiseizure effects in the PTX-induced epilepsy model. Additional studies with vitexin demonstrated that though it did not suppress spontaneous seizure behaviors in our genetic model of epilepsy, it did significantly inhibit PTZ-induced electrographic activity. These findings support the continued exploration of the translational potential of the vitexin scaffold. This work advances our search for safer, more effective antiseizure drugs and could pave the way for vitexin-based treatments for epilepsy and related disorders.

## Graphical Abstract

**Figure:**
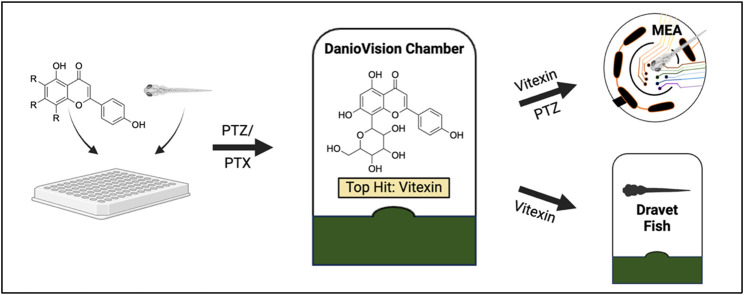
Created in BioRender.

## Introduction

Epilepsy is a chronic brain disorder affecting around 65 million people of all ages worldwide. It is marked by spontaneous, recurrent seizures, with individuals also experiencing debilitating somatic and psychiatric comorbidities. Antiseizure medicines (previously referred to as antiepileptic drugs) serve as the first line of treatment and this armamentarium comprises of over 30 FDA approved drugs. This includes first generation medications such as valproic acid, carbamazepine and phenobarbital, to the newer third generation drugs like lacosamide (Hanaya and Arita, 2016; Sánchez et al., 2024). Though many have proven to be successful in alleviating symptoms, drug resistance is observed in ∼30% of patients (Kwan and Brodie, 2000). Additionally, some of these antiseizure medicines carry the potential of serious adverse effects, including gastrointestinal effects, psychotic episodes, depression and osteoporosis (Seidenberg et al., 2009). There is therefore need for novel treatment approaches that can provide safer and more effective alternatives for persons with epilepsy, which will ultimately improve their quality of life.

A rapidly growing body of research substantiates a critical role for oxidative stress in the pathogenesis of epilepsy. Oxidative stress arises from an imbalance where the production of reactive oxygen/nitrogen species exceeds their elimination via intrinsic antioxidant mechanisms. Increased levels of these “pro-oxidants” can damage macromolecules, such as nucleic acids, proteins and lipids, and hence destroy cellular structure and function (Aguiar et al., 2012). The high lipid content and oxygen consumption rate of the brain make it especially susceptible to oxidative damage (Cobley et al., 2018). Human studies have revealed an increased oxidative state in the brains and blood of patients with epilepsy, as marked by depletion of the antioxidants such as GSH (reduced glutathione), vitamin E and catalase (Mueller et al., 2001; Menon et al., 2014). Similar shifts in redox equilibrium have been identified in multiple rodent models of epilepsy (pilocarpine-induced, kainate-induced, amygdala kindling, electroconvulsions, PTZ/PTX-induced) (Łukawski and Czuczwar, 2023). Mice lacking manganese superoxide dismutase 2, a mitochondrial antioxidant enzyme have heightened unprovoked and induced seizure susceptibility (Liang and Patel, 2004; Liang et al., 2012). This implicates oxidative stress and mitochondrial dysfunction as causal forces in epileptogenesis. Consequently, multiple groups have already started to explore the benefits of antioxidants in disrupting the development and progression of epilepsy (Liang et al., 2012).

Flavonoids are secondary metabolites found in plants that form a part of their stress response armory and are well known for their antioxidant and anti-inflammatory actions (Roy et al., 2022). Flavonoids can (i) scavenge free radicals by stabilizing the reactive oxygen species, (ii) inhibit nitric-oxide (NO) synthase and thus reduce NO production (NO can react with free radicals to form the highly toxic species, peroxynitrite) and (iii) inhibit xanthine oxidase activity, a source of oxygen free radicals (Nijveldt et al., 2001). In the context of brain disorders, flavonoids are particularly attractive as many readily cross the blood brain barrier (Youdim et al., 2003). Vitexin, an apigenin flavone glycoside, falls into this category. It is naturally found in plants such as passion flowers, mung bean, hawthorn, and bamboo (Ranjan et al., 2023). Vitexin has been shown to suppress Na^+^-K^+^-Cl^−^ co-transporter 1 expression, improve blood brain barrier integrity and ultimately reduce seizure susceptibility following hypoxic ischemia in neonatal brains of Sprague Dawley rats (Luo et al., 2018). De Oliviera et al., found that vitexin blocked pentylenetetrazole (PTZ)- and picrotoxin (PTX)-stimulated seizure activity and showed anxiolytic properties in Wistar rats (de Oliveira et al., 2020). This group further showed chronic exposure to vitexin also suppresses PTZ-induced kindling (de Oliveira et al., 2024). Closely related flavone glycosides also show antioxidant and anti-inflammatory activity (Wei et al., 2014; He et al., 2016; Wang et al., 2022; Kantharaj et al., 2023), and thus additional studies into the antiseizure effects of vitexin and related compounds are warranted.

Zebrafish serve as a useful model organism to study epilepsy as they exhibit the hallmark feature of the disorder, i.e., seizures. They share a high degree of genetic similarity with humans and many signal transduction and development pathways are conserved. Zebrafish can be easily employed for large scale drug screening as adults generate large clutches externally, offspring undergo rapid development and larvae are small enough for drug testing using a 96-well format (Chakraborty et al., 2009). To date, drug candidates (including antiseizure compounds) found in preclinical zebrafish screens have advanced to compassionate use and clinical trials (Baraban, 2021; Patton et al., 2021). Vitexin has already shown neuroprotective effects in zebrafish, suppressing acrylamide-induced neuroinflammation and improving antioxidant capacity (Krishnan and Kang, 2019). Here, we further explore the antiseizure properties of vitexin and structurally similar compounds (isovitexin, vitexin 2-O-rhamnoside, vitexin-4-O-glucoside and saponarin) using chemically-induced and genetic zebrafish models of epilepsy.

## Materials and methods

### Compounds

#### Source

Vitexin (CAS Number: 3681-93-4; purity degree ≥98%), isovitexin (CAS Number: 38953-85-4; ≥98%), vitexin 2-O-rhamnoside (CAS Number: 64820-99-1; ≥95%), vitexin-4-O-glucoside (CAS Number: 178468-00-3; ≥95%), saponarin (isovitexin 7-glucoside, CAS Number: 20310-89-8; ≥95%) and stiripentol (CAS Number: 49763-96-4; ≥98%) were purchased from Cayman Chemical Company, MI, United States. The proconvulsant drugs pentylenetetrazole (PTZ) (CAS Number: 54-95-5; ≥98%) and picrotoxin (PTX) (CAS Number: 124-87-8; ≥98%) were purchased from Sigma Aldrich, MO and Cayman Chemical Company, MI, United States, respectively. Dimethyl sulfoxide (DMSO) (CAS Number: 67-68-5) was purchased from VWR, OH.

#### Preparation

Stocks solutions (25 or 50 mM) of vitexin, isovitexin, vitexin 2-O-rhamnoside, vitexin-4-O-glucoside, saponarin and stiripentol were made in DMSO. All stocks were stored at −20°C for ≤3 months and working solutions were made in egg water (0.03% Instant Ocean and 0.0002% methylene blue in reverse-osmosis distilled water) within an hour of each experiment (final concentration of DMSO <2%). Drug solutions of PTZ and PTX were freshly prepared in egg water prior to treatment steps.

### Zebrafish maintenance

All protocols and experiments were in line with National Institutes of Health and the University of Richmond guidelines. Experiments were conducted after approval by the Institutional Animal Care and Use Committee (protocol #21-05-001). Adult male and female AB strain zebrafish were purchased from the Zebrafish International Resource Center (ZIRC, Cat ID ZL86). *Scn1lab* ^*+/−*^ zebrafish were generously gifted by Dr. Scott Baraban at the University of California, San Francisco. Zebrafish were maintained in a closed-water system with controlled conditions (temperature: 27°C–29°C; pH: 7–7.5; conductivity (EC): 680-730 μS/cm) in a facility under a 14:10 h light/dark schedule (8:00 a.m.-10:00 p.m. ET). Breeders were fed twice a day with commercial fish flake food (Tetramin tropical flakes) and Zeigler adult (1 mm) pellets in the morning and flake food, pellets and live brine shrimp in the afternoon. Male and female zebrafish were bred in a 1:1 or 2:1 ratio and the resulting embryos were incubated at 28°C ± 0.5°C in egg water. All experiments were conducted at 5 days post-fertilization (dpf) and larvae were randomly assigned to treatment groups. At this stage, larvae cannot be sexually distinguished.

### Evaluating anti-seizure properties of compounds

#### Behavioral assays

All behavioral activity was captured using a DanioVision system running EthoVision XT 15 software (DanioVision, Noldus Information Technology). Videos were recorded at 25 frames/sec and the detection settings were as previously reported (Griffin et al., 2021) (method; DanioVision, sensitivity; 110, video pixel smoothing; low, track noise reduction; on, subject contour; 1 pixel (contour dilation, erode first then dilate), subject size; 4-4065).

#### Chemically-induced seizure assay

Zebrafish larvae were transferred to a clear, flat bottomed 96 well-microplate followed by subsequent treatment with 0-500 µM vitexin or related glycosides and 25 µM stiripentol (one larva *per* well, 4-6 larvae *per* concentration). The plate was transferred to the DanioVision observation chamber and left undisturbed for 45 min to allow for acclimation. A 15-min recording was then taken to quantify basal activity. This also served as a 1-h pretreatment before exposure to the proconvulsant drugs. To induce seizures, larvae were then treated with 10 mM PTZ or 1 mM PTX and after 30 min, behavior was tracked for 15 min. Test compound concentrations were maintained at 1X throughout this treatment period. Immediately after the experiment, toxicity was assessed by monitoring heart rate and larval movement under a microscope. Experiments were repeated using 3-4 independent AB zebrafish clutches, yielding sample sizes of >12 fish per treatment group, in alignment with established standards for statistical rigor (Baraban, 2013; Griffin et al., 2017; Griffin et al., 2020; Habjan et al., 2024).

#### Genetically-induced seizure assay

*Scn1lab* ^*+/−*^ zebrafish breeders were in-crossed and on day 5, *scnl1ab* ^*−/−*^ mutant larvae (identified via black pigmentation (Baraban et al., 2013)) were selected for the experiment. These larvae were transferred to a clear, flat bottomed 96 well-microplate and were treated with 0–1,000 µM vitexin or 100 µM stiripentol. The plate was transferred to the DanioVision observation chamber for a 30-min acclimation/treatment window and then 15-min recordings were taken to quantify antiseizure activity. All larvae were monitored after the experiment to identify any heart rate or movement changes that could signify toxicity. The experiment was repeated with three different clutches.

#### Electrophysiology

The Axion Maestro system was utilized to perform multielectrode array (MEA) electrophysiology studies. Low melting agarose (1.5%) was dissolved in egg water and kept on a heating block set at 45°C. Five dpf larvae were then incubated with either DMSO control, 500 µM vitexin, or 25 µM stiripentol for 1 h. All the experiments were conducted on 6-well CytoView MEA plates (Axion BioSystems- M348-Tmea-6B) with 64 poly (3,4-ethylenedioxythiophene) polystyrene sulfonate electrodes *per* well. To plate the larvae, a pipette was used to gently transfer each larva to the CytoView wells (one larva *per* well). Larvae were then treated with a paralytic, pancuronium, to ensure recording data was not skewed by physical movements of the zebrafish larvae. Once the larvae were completely still, cooled 1% agarose solution was added to the well and an eyelash tool was then used to correctly orient the fish over the electrodes until the agarose solidified. This step was performed using a microscope to ensure the larvae had their dorsal side down and were flat against the electrodes to ensure optimal electrode coverage.

The CytoView plate was transferred to the recording chamber of the Axion Biosystems Maestro Volt to capture real-time, spontaneous neural activity. After 2 min in the chamber, larvae were treated with either egg water, 10 mM PTZ, 10 mM PTZ +500 µM vitexin or 10 mM PTZ +500 µM stiripentol, respectively, and activity was tracked for another 2 min. AxIS 3.12.2 software was used to acquire and process the data. Acquisition settings: Spike Detector was set at 6 x STD and Mean Firing Rate Estimation was set at 10 s. Once the experiment was complete, larvae were inspected under the microscope to confirm that larval positioning did not change during the experiment and to identify the numbered electrodes that were in contact with the larval head (brain). Although the larval head typically covered 2–3 electrodes on the CytoView array, recordings were analyzed from a single electrode directly contacting the optic tectum/midbrain region that exhibited the most stable and robust signal.

### Statistical analysis

Statistical analyses were performed using GraphPad Prism (version 10.0, GraphPad Software, United States). Normality was assessed by applying either the Shapiro-Wilk or D’Agostino-Pearson tests, depending on sample size. Datasets that did not follow a normal distribution were analyzed with the Kruskal-Wallis test with Dunn’s post hoc test, while all others were analyzed using one-way ANOVA followed by Dunnett’s multiple comparisons test. Experimental data were expressed as mean ± SEM. Significant differences were represented as follows: *p ≤ 0.05; **p ≤ 0.01; ***p ≤ 0.001; ****p ≤ 0.0001.

## Results

### Impact of vitexin and related flavone glycosides on basal activity

**FIGURE 1:**
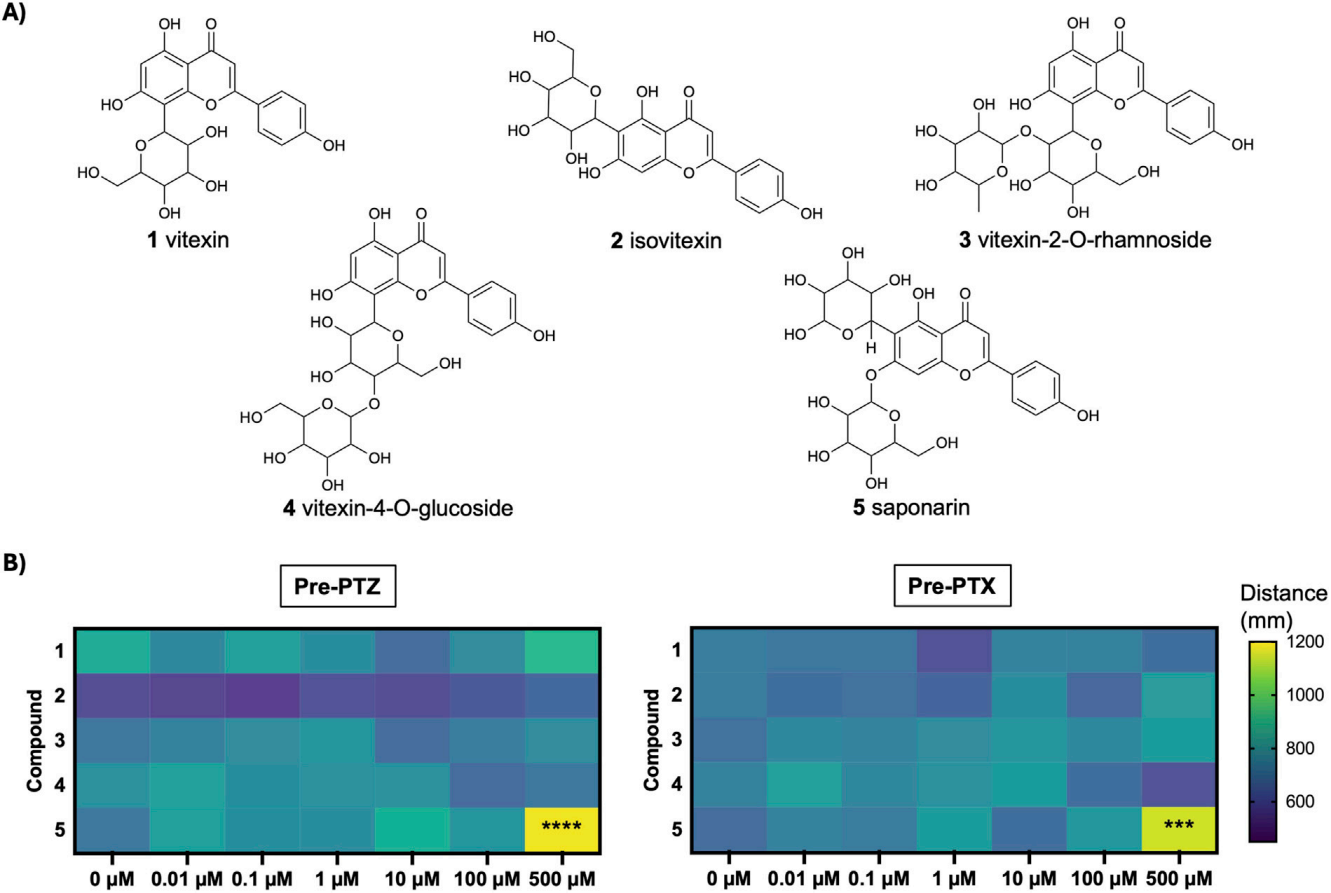
Structure and basal effects of flavones under investigation. **(A)** Chemical structures of compounds: vitexin (1), isovitexin (2), vitexin-2-O-rhamnoside (3), vitexin-4-O-glucoside (4), saponarin (5). **(B)** Heat map depicts mean movement (distance, mm) after 5 dpf larvae were treated with 0, 0.1, 1, 10, 100, 500 µM of compounds 1–5, prior to exposure to PTZ or PTX. N = 15–29 larvae per concentration. Analyses For 500 µM saponarin (5) vs. 0 μM control: Pre-PTZ, F_(6, 118)_ = 5.99, ****p < 0.0001 (one-way ANOVA with Dunnett’s test); Pre-PTX, ***p < 0.001 (Kruskal–Wallis with Dunn’s test).

The chemical structures of the flavones under investigation are all illustrated in Figure 1A. In order to evaluate whether these compounds affected normal swim behavior, larval zebrafish were exposed to varying concentrations (0-500 µM) for 45 min and locomotor activity was measured as the total distance moved over a 15-min recording. As shown in the heat map in Figure 1B, there were no significant differences in baseline swimming behavior when the larvae were in the presence of vitexin (1), isovitexin (2), vitexin 2-O-rhamnoside (3) or vitexin-4-O-glucoside (4). Saponarin (5) was the only compound to alter basal movement and this was only observed at 500 µM. Specifically, it caused a significant increase in total distance moved as compared to the vehicle (0 µM saponarin) treatment (*p* ≤ 0.001). This hyperactivity did not appear to be seizure-like, since stage II and III events (Baraban et al., 2005) were not observed during manual video analyses.

### Vitexin and isovitexin show protective effects against chemically-induced behavioral seizures

**FIGURE 2:**
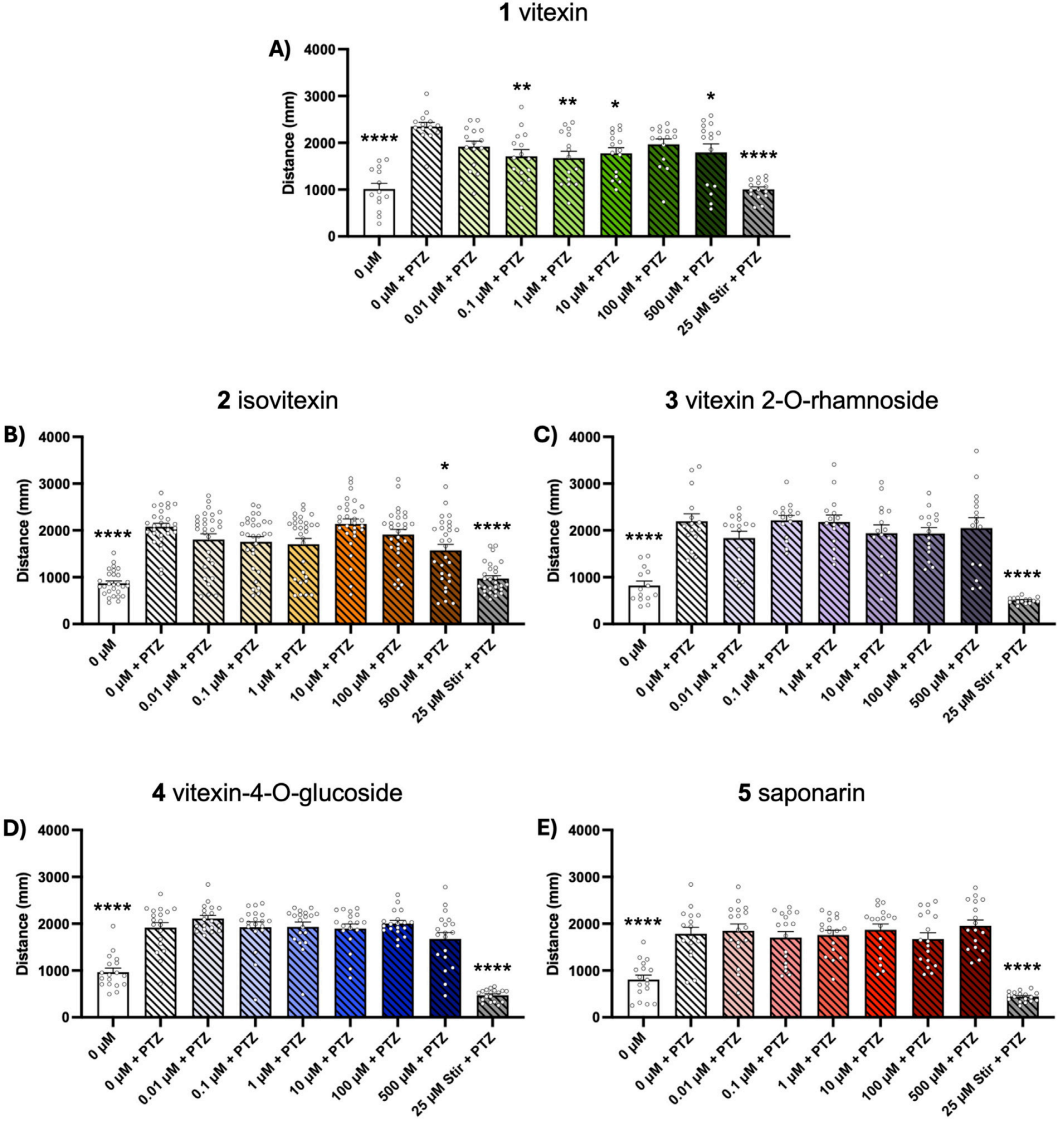
Dose-response effects of compounds 1–5 on PTZ-induced seizure activity. Five dpf zebrafish larvae were treated with 0-500 µM of each compound and 50 µM stiripentol (stir) for 1 h followed by a 15-min exposure to 10 mM PTZ. Behavior was then tracked for 15 min and total distance traveled was extracted to assess seizure activity. Bar graphs represent the mean ± SEM (n = 14–29 per group). **(A,C,E)** One-way ANOVA: F_(8, 124)_ = 11.64; F_(8, 125)_ = 18.70; F_(8, 143)_ = 20.14 (all *p* < 0.0001), with Dunnett’s post hoc test: *p < 0.05, **p < 0.01, ****p < 0.0001 vs. 0 μM vitexin + PTZ control. **(B,D)** Kruskal–Wallis with Dunn’s post hoc test: *p < 0.05, ****p < 0.0001 vs. 0 μM vitexin + PTZ control.

**FIGURE 3:**
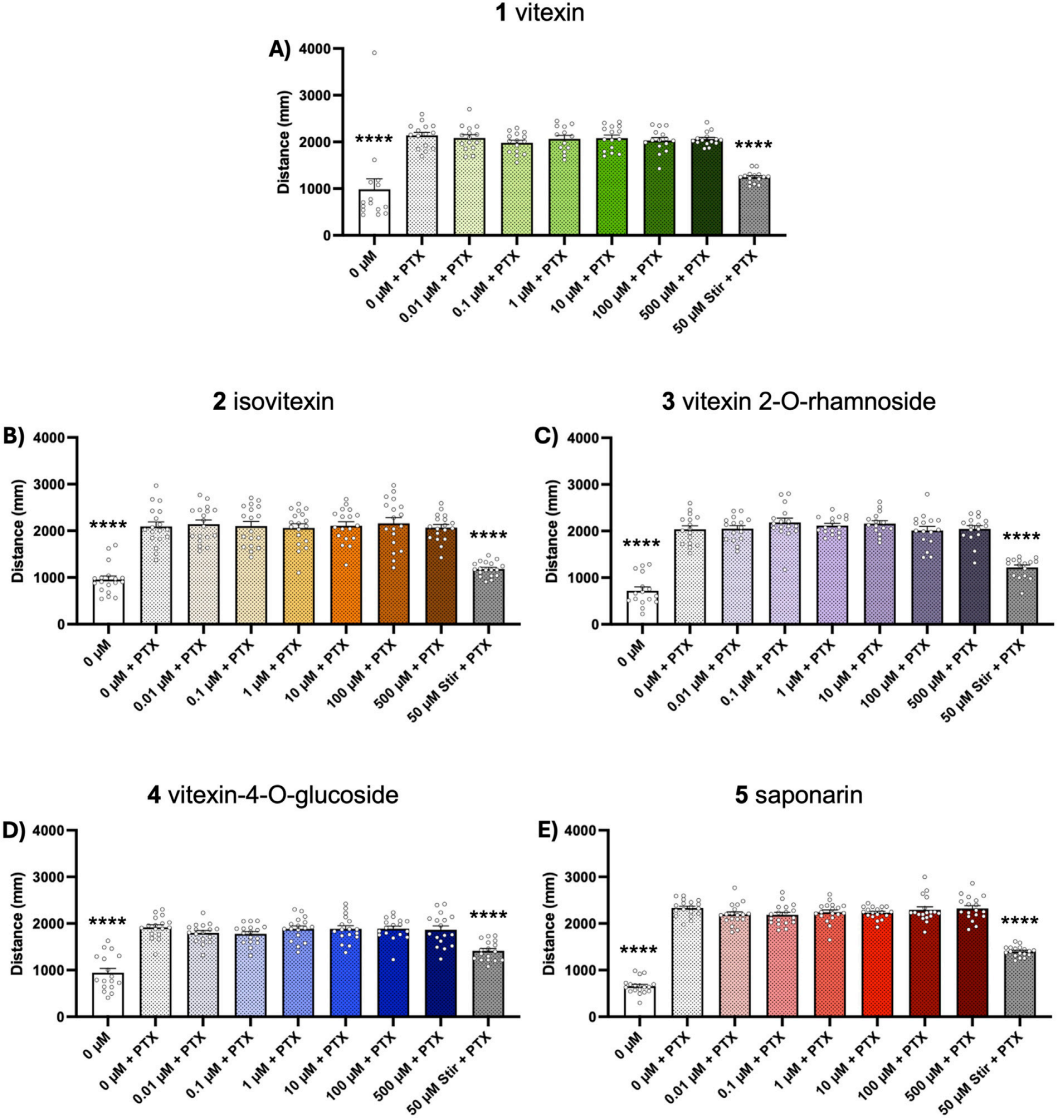
Dose-response effects of compounds 1-5 on PTX-induced seizure activity. Five dpf zebrafish larvae were treated with 0-500 µM of each compound for 1 h followed by a 15-min exposure to 1 mM PTX. Behavior was then tracked for 15 min and total distance traveled was extracted to assess seizure activity. Bar graphs represent the mean ± SEM (n = 13–18 per group). **(A)** Kruskal–Wallis with Dunn’s post hoc test: ****p < 0.0001 vs. 0 μM vitexin + PTX control. **(B–E)** One-way ANOVA: F_(8, 153)_ = 27.28; F_(8, 134)_ = 48.35; F_(8, 144)_ = 24.71; F_(8,153)_ = 142.8 (all *p* < 0.0001). Post hoc Dunnett multiple comparison test, ****p < 0.0001 vs. 0 µM vitexin + PTX control.

Chemically-induced seizure models are widely used as tools for early-stage assessment of potential anticonvulsant compounds. Here, we evaluated the ability of our test compounds to reduce behavioral seizures induced by 10 mM PTZ and 1 mM PTX in 5-day old zebrafish larvae. We found that 1-h pretreatment with vitexin (1) inhibited seizure activity in the PTZ assays, as indicated by total swim distance (*p* ≤ 0.05, Figure 2A). Although not effective at lower concentrations, isovitexin (2) significantly reduced induced seizure behaviors at 500 µM (*p* ≤ 0.05, Figure 2B). In contrast, vitexin 2-O-rhamnoside, vitexin-4-O-glucoside and saponarin (3-5) did not affect PTZ-stimulated behavioral seizures (Figures 2C–E). Interestingly, none of the compounds under investigation modified behavioral seizures generated after PTX exposure (Figures 3A–E). The antiseizure medication, stiripentol, worked effectively as a positive control and suppressed seizures in both chemically-induced seizure models.

### Vitexin does not augment spontaneous seizures in Dravet zebrafish model

**FIGURE 4:**
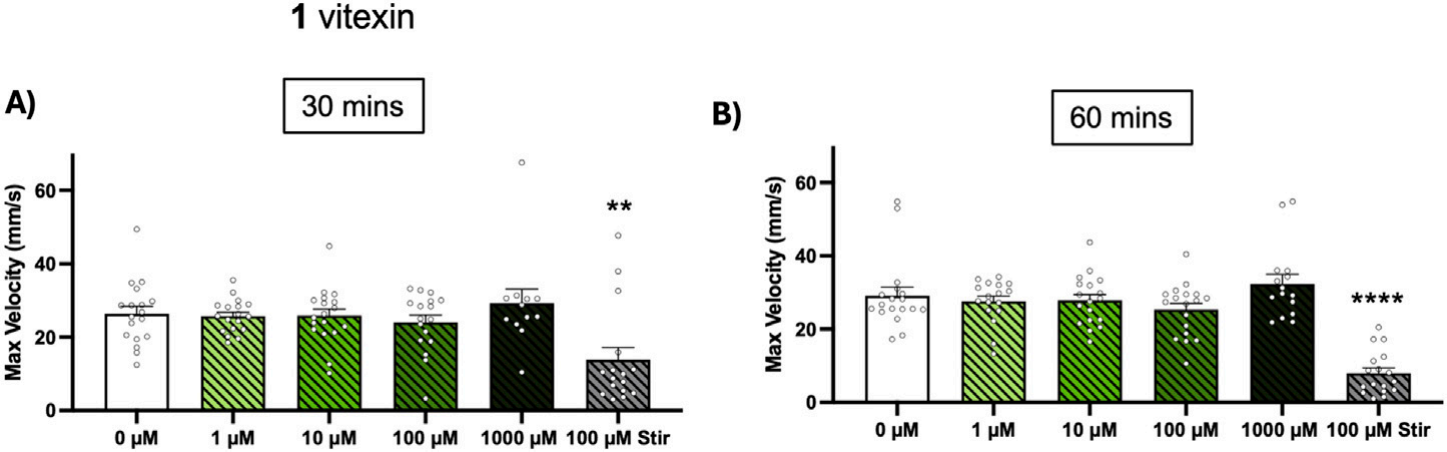
Effect of vitexin (1) on spontaneous seizures in zebrafish DS model. *Scn1lab* ^*+/−*^ breeders were in-crossed and *scn1lab* ^*−/−*^ larvae were identified at 5 dpf via their black pigmentation. In 96 well-plates, the larvae were exposed to 0–1,000 µM of vitexin or 100 µM stiripentol (stir) and 15-min recordings were taken at **(A)** t = 30 min and **(B)** t = 60 min. Seizure behavior was quantified from maximum velocity measurements. Bars are shown as mean ± SEM (n = 12–18 per concentration). Kruskal-Wallis with Dunn’s multiple comparisons test: **p < 0.01 and ****p < 0.0001 (compared to 0 µM treatment).

With vitexin (1) exhibiting promising anticonvulsant effects over a wide range of concentrations, we aimed to investigate whether this effect could also be observed in other models of epilepsy. We employed the zebrafish model of Dravet Syndrome, *scn1lab* ^*−/−*^ larvae, to determine if vitexin could inhibit spontaneous seizures in this genetic model of epilepsy. In humans, the disorder is linked to haploinsufficiency in its homolog, *SCN1A*, which encodes for the alpha subunit of Na_V_1.1 voltage-gated sodium channels. *Scn1lab* ^*−/−*^ zebrafish exhibit electrographic seizures at 3 dpf and striking tonic-clonic like behavioral seizures by 5 dpf. Unlike in the chemically-induced models, seizures were quantified through maximum velocity measurements since total swim distance has been reported as an unreliable seizure marker in these larvae (Griffin et al., 2021). We found that up to 1,000 µM vitexin (1) did not augment behavioral seizures while stiripentol (approved for treatment of Dravet Syndrome in children 6 months and older), did (Figures 4A,B).

### Vitexin inhibits electrographic seizure-like activity of PTZ-exposed zebrafish

**FIGURE 5:**
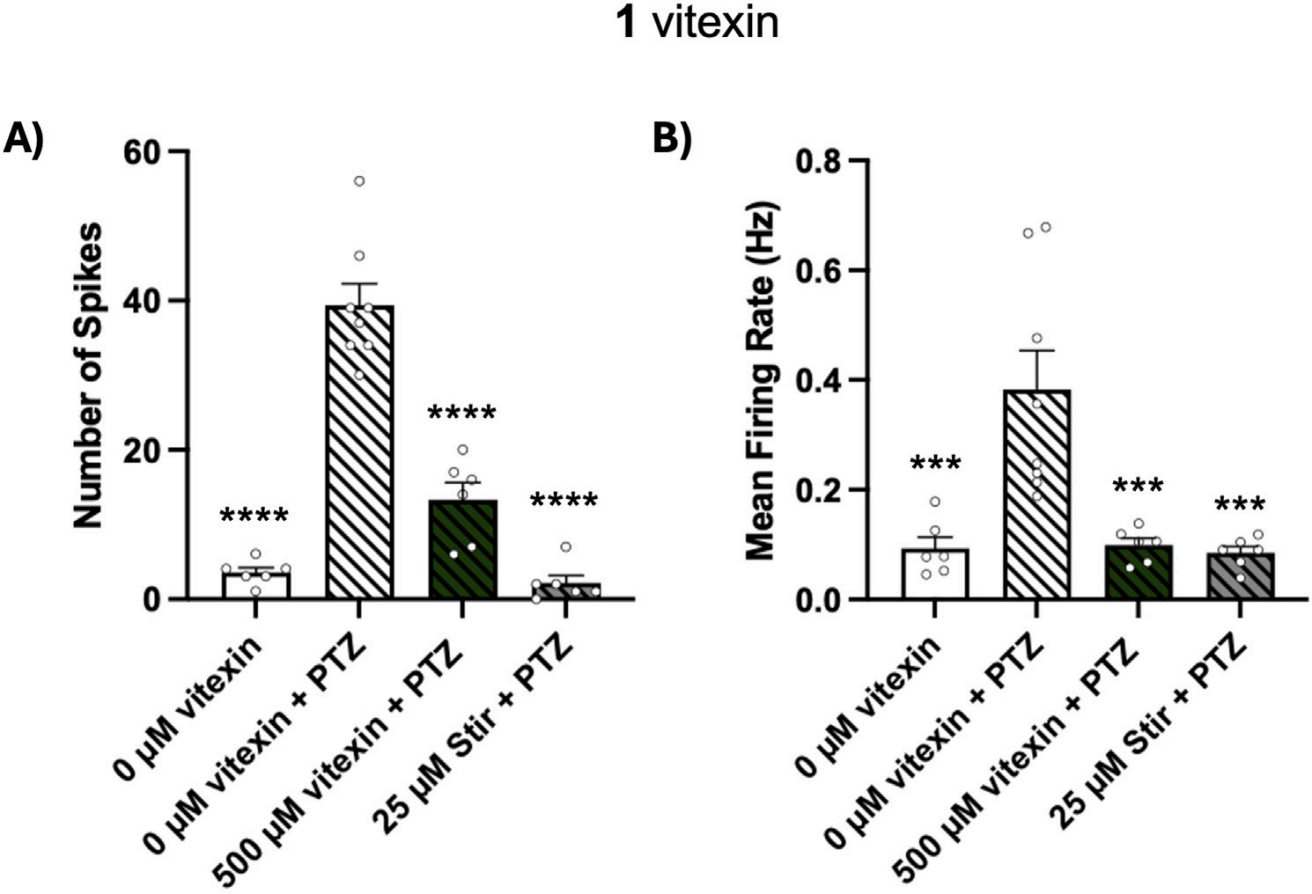
Effect of vitexin (1) on PTZ-induced electrographic events. Larvae were treated with 0 µM vitexin (1% DMSO), 500 µM vitexin or 25 µM stiripentol (stir) for 1 h and then embedded in separate wells of the MEA plate. Egg water or 10 mM PTZ was then added to achieve the following four conditions: 0 µM vitexin (N = 6), 0 µM vitexin + PTZ (N = 8), 500 µM vitexin + PTZ (N = 6), 25 µM stir + PTZ (N = 6). **(A)** Number of spikes and **(B)** mean firing rate were quantified using the Axion MEA system. Data are represented in bar graphs as mean ± SEM. One-way ANOVA with Post hoc Dunnett multiple comparison test: spike; F_(3, 22)_ = 70.48, ****p < 0.0001 and firing rate; F_(3, 22)_ = 11.14, ***p < 0.001 (compared to 0 µM + PTZ treatment).

Since vitexin (1) blocked behavioral seizures in our PTZ assay, we next measured whether it could block PTZ-stimulated electrographic events in 5 dpf larvae. We tested 500 μM, the highest concentration of vitexin used in our behavioral tests. We quantified seizure-like activity through two parameters recorded from the MEA device: number of spikes and mean firing rate. One hour pretreatment with 500 µM vitexin significantly blocked PTZ-stimulated increases in these measures, Figure 5A spikes: *p* ≤ 0.0001 and Figure 5B firing rate: *p* ≤ 0.001. Similar reductions were observed in fish treated with the positive control, stiripentol. Thus, using zebrafish, we have not only confirmed vitexin’s inhibitory effects on behavioral manifestations of seizures but have shown its ability to block seizure-like activity at the brain level.

## Discussion

Plants have served as a rich source of bioactive molecules for drug discovery and recent developments in analytical technologies have revived research in this area. Particularly noteworthy is the 2018 FDA approval of the first ever plant-derived medication for epilepsy, Epidiolex. Indicated for Dravet and Lennox Gastaut Syndrome, Epidiolex is a purified cannabidiol solution extracted from cannabis plants. This success underscores the importance of revisiting natural product sources in the context of treatment-resistant disorders like epilepsy. In the present study, we used a handful of zebrafish epilepsy models to assess the antiseizure potential of vitexin and vitexin-related flavones, which are all commonly found in traditional herbal Chinese medicines.

We employed two GABA_A_ receptor antagonists, PTZ and PTX, to induce acute seizures in larval zebrafish. PTZ rodent seizure models have long been used by the NIH for its anticonvulsant screening program (Yuen and Trocóniz, 2015). The PTZ zebrafish acute seizure model was first described by Baraban and colleagues in 2005 and has been used to recapitulate features of generalized tonic-clonic and absence seizures (Baraban et al., 2005; Baraban et al., 2007; D'Amora et al., 2023). Similar to PTZ, PTX has also risen as a reliable chemoconvulsant in wild type zebrafish lines and rodents, and has found utility in recent antiseizure drug efficacy studies (Wong et al., 2010; Randrianarivo et al., 2016; Golovenko et al., 2019; Bandara et al., 2020; Balamurugam and Lakshmanan, 2022). We began this study by first evaluating the effectiveness of vitexin, isovitexin, vitexin 2-O-rhamnoside, vitexin-4-O-glucoside and saponarin against behavioral seizures induced by PTZ and PTX respectively. Aside from the increased basal locomotion observed with 500 µM saponarin treatment, no other concentrations of saponarin or any of other compounds displayed undesirable or toxic effects in the larvae. Both PTZ and PTX significantly increased distance moved by the 5 dpf larvae, which is a widely accepted readout of seizure activity and severity in these models. PTZ-induced effects were inhibited by both vitexin and isovitexin. Though we noted inhibition across various concentrations of vitexin, isovitexin only displayed efficacy at the highest concentration tested (500 µM). Neither showed anti-seizure activity in our PTX model.

The inclusion of both PTZ and PTX facilitated comparison with prior murine seizure studies and assessment of whether zebrafish responses mirrored known mammalian outcomes. There was one striking difference: unlike in zebrafish, vitexin blocked the effects of both GABA_A_ receptor antagonists in mice. Specifically, vitexin significantly reduced PTZ- and PTX-induced seizures in addition to increasing the latency to the first seizure. Vitexin failed to protect against seizures induced by kainic acid, a glutamate receptor agonist, and therefore implied its underlying mechanism of action could be via GABA neurotransmission modulation (de Oliveira et al., 2020). As GABA_A_ receptor antagonists, PTZ and PTX have distinct (though overlapping) binding patterns and dynamics at these proteins (Huang et al., 2001). They also have other CNS targets. PTX interacts with GABA_C_, glycine and glutamate-gated Cl^−^ channels and both compounds bind to 5-HT3A receptors (Schmieden et al., 1989; Zhang et al., 1995; Etter et al., 1999; Das et al., 2003). The unexpected PTZ bias shown by both vitexin and isovitexin in our zebrafish studies may reflect actions mediated by these additional target sites and/or potential species-specific variations in related proteins. Nevertheless, given the prolific use of PTZ over PTX for seizure induction and antiseizure drug screening (Johan Arief et al., 2018), the efficacy of vitexin in the PTZ-zebrafish model further implicated its clinical relevance.

Since vitexin displayed more potent inhibitory effects when compared to isovitexin, inhibiting seizure activity at concentrations as low as 0.1 µM, we decided to solely focus on assessing the antiseizure properties of vitexin in our other zebrafish assays. For this, we first turned to larvae lacking functional *scn1lab*, the zebrafish model of Dravet Syndrome. Mounting evidence point to roles for metabolic dysfunction and redox imbalance in the pathogenesis of the disorder. Dimercaprol, approved for arsenic and mercury poisoning, was recently shown to effectively reduce seizures in Dravet zebrafish. This was tied to its ability to enhance antioxidant capacity by increasing intracellular GSH concentrations (Sri Hari et al., 2023). Ketogenic diets also elevate *in vivo* GSH levels and their clinical incorporation have produced positive effects in helping patients with Dravet Syndrome manage their symptoms (Yu et al., 2023). As an antioxidant, vitexin has been shown to increase GSH/GSSG (reduced/oxidized glutathione) ratio in addition to other protective effects (Babaei et al., 2020; Song et al., 2023). Nonetheless, we found that vitexin was not effective in reducing unprovoked seizure behaviors in *scn1lab* ^*−/−*^ zebrafish. It is important to note that all our studies were done with acute vitexin treatments (∼1 h) but studies with dimercaprol utilized more extended treatment schedules, suggesting future work with longer treatment schedules is necessary.

The “gold standard” in the study of epilepsy across all species, including humans, is to monitor electrographic activity in the brain (i.e., electroencephalography or EEG). Furthermore, numerous studies have effectively employed single electrode *in vivo* electrophysiology to obtain local field potential recordings from larval zebrafish (Baraban et al., 2005; Hortopan et al., 2010; Afrikanova et al., 2013; Baraban, 2013; Pena et al., 2017; Zabinyakov et al., 2017; Griffin et al., 2021). This method revealed the antiseizure potential of drugs such as fenfluramine and lorcaserin, findings later substantiated by human studies (Dinday and Baraban, 2015; Griffin et al., 2017; Tolete et al., 2018). In 2020, fenfluramine was FDA approved to treat seizures associated with Dravet Syndrome in individuals 2 years or older. Though single electrode electrophysiology in zebrafish is minimally invasive, it is laborious and low throughput. Here we aimed to capture brain electrical activity using a non-invasive, MEA system. This device was previously validated with the first-generation antiseizure drug valproate and was also adopted to capture the seizure-suppressing effects of dimercaprol (Tomasello and Sive, 2020; Sri Hari et al., 2023). Our objective was to assess whether the ability of vitexin to mitigate PTZ-induced behavioral manifestations also extended to the attenuation of PTZ-evoked electrographic events. Through our work we demonstrated that 1-h pretreatment with 500 µM vitexin attenuates increases in the number of spikes and mean spike rate induced by PTZ exposure. This reduction was similar to what we observed when larvae were pretreated with the already approved drug, stiripentol. MEAs as a platform for epilepsy drug discovery efforts does need further validation and we hope to also compare the therapeutic effects of vitexin on zebrafish monitored through single electrode LFP configurations, since the latter has reported predictive validity for clinical outcomes.

Taken together, our findings reinforce that vitexin shows significant promise as a drug candidate for epilepsy. An important avenue for future studies is to employ neurochemical analyses to quantify whether vitexin modulates brain oxidative status in our model. Establishing this link could help clarify the extent to which this mechanism contributes to its antiseizure properties. Additionally, these experiments may provide insights into broader clinical applications of this flavonoid. Chronic use of some antiseizure medicines, particularly first-generation drugs like valproate and phenytoin, may lead to increased reactive oxygen species generation. This has been implicated as a driver of pharmaco-resistance in epilepsy (Martinc et al., 2012). Therefore, while this study focuses on vitexin as a monotherapy, the compound may also prove beneficial as an adjunctive therapy for epilepsy. Comprehensive pharmacokinetic studies would be warranted to assess potential drug-drug interactions. Comorbidities are also a significant burden to patients with epilepsy and many times are either not addressed by antiseizure drugs or in some cases are worsened by these drugs. In rodents, vitexin administration reduced anxiety-related behaviors and also reversed scopolamine-induced memory impairment (Abbasi et al., 2013; de Oliveira et al., 2020). These studies imply vitexin could help patients manage their comorbid symptoms as well. Although isovitexin was not investigated as extensively, the current study provides evidence of its antiseizure properties. This activity was absent in the other related flavones and emphasizes the need for more detailed structure-activity relationship studies to understand the key functional groups responsible for the reported seizure-reducing effects.

## Conclusion

As our understanding of the role of oxidative stress and inflammation across various epilepsies expands, explorations of the antiseizure effects of antioxidants are following suit. This study supports the continued preclinical and clinical investigations of vitexin and its scaffold for epilepsy treatment. We used zebrafish to rapidly screen compounds with similar chemical moieties, employing behavioral and electrophysiology readouts to quantify translational potential. While this teleost model cannot fully recapitulate all the pharmacokinetic and pharmacodynamic complexities of mammals, multi-stage screening in zebrafish has already shown success and we hope will continue to reveal novel candidates for epilepsy drug development and beyond.

## Data Availability

The raw data supporting the conclusions of this article will be made available by the authors, without undue reservation.
